# WHAT MAKES A BEAUTIFUL DAY IN THE NEIGHBORHOOD? PLACE AS A CONTRIBUTOR TO FUNCTION IN LATER LIFE

**DOI:** 10.1093/geroni/igac059.1009

**Published:** 2022-12-20

**Authors:** Andrea Rosso

## Abstract

Neighborhood environments are increasingly recognized as an important determinant of health and function in older adults. Environmental supports such as density of intersections and available community resources can promote activity and participation which in turn promotes physiological health. In contrast, barriers such as disorder and high traffic can limit activity and participation, particularly for those at high risk for mobility limitations and falls. Here, we present five papers exploring these relations. First, Kate Duchowny presents work assessing relations of the built and social environment with muscle strength in the Health and Retirement Study. Two papers utilizing walkability assessments using Google Street View in a physical activity intervention trial are presented; Kyle Moored demonstrates relations of neighborhood walkability with Global Positioning System (GPS)-derived time out of home and Anisha Suri assesses how the relation between actigraphy-derived gait quality and daily step counts differs by walkability. Next, Philippa Clarke presents data on the association of neighborhood environment with diabetes risk in those with low visual function in an administrative claims database. Finally, Pam Dunlap describes results of a systematic review of outdoor environmental risk factors for falls and fear of falling. Together, these papers will demonstrate the breadth of ways in which neighborhood environments and function relate to determine health outcomes for older adults.

